# A genomic overview of the population structure of *Salmonella*

**DOI:** 10.1371/journal.pgen.1007261

**Published:** 2018-04-05

**Authors:** Nabil-Fareed Alikhan, Zhemin Zhou, Martin J. Sergeant, Mark Achtman

**Affiliations:** Warwick Medical School, University of Warwick, Coventry, United Kingdom; Universidad de Sevilla, SPAIN

## Abstract

For many decades, *Salmonella enterica* has been subdivided by serological properties into serovars or further subdivided for epidemiological tracing by a variety of diagnostic tests with higher resolution. Recently, it has been proposed that so-called eBurst groups (eBGs) based on the alleles of seven housekeeping genes (legacy multilocus sequence typing [MLST]) corresponded to natural populations and could replace serotyping. However, this approach lacks the resolution needed for epidemiological tracing and the existence of natural populations had not been independently validated by independent criteria. Here, we describe EnteroBase, a web-based platform that assembles draft genomes from Illumina short reads in the public domain or that are uploaded by users. EnteroBase implements legacy MLST as well as ribosomal gene MLST (rMLST), core genome MLST (cgMLST), and whole genome MLST (wgMLST) and currently contains over 100,000 assembled genomes from *Salmonella*. It also provides graphical tools for visual interrogation of these genotypes and those based on core single nucleotide polymorphisms (SNPs). eBGs based on legacy MLST are largely consistent with eBGs based on rMLST, thus demonstrating that these correspond to natural populations. rMLST also facilitated the selection of representative genotypes for SNP analyses of the entire breadth of diversity within *Salmonella*. In contrast, cgMLST provides the resolution needed for epidemiological investigations. These observations show that genomic genotyping, with the assistance of EnteroBase, can be applied at all levels of diversity within the *Salmonella* genus.

## Introduction

*S*. *enterica* has long been subdivided by differential antibody reactions into what are now called serovars [1]. The use of specific antibodies that could identify distinct cell-surface antigens within lipopolysaccharide and flagella have led to the distinction of >2,500 serovars that differ in their antigenic formulas [2]. Some of these serovars cause invasive diseases of specific hosts, including the human-specific typhoidal serovars such as Typhi, Paratyphi A, and Paratyphi C, whereas others cause gastroenteritis of varying severity in multiple hosts (see Fig 1 in [3]). *Salmonella* is also subdivided taxonomically into *S*. *enterica*, which contains multiple subspecies, and a separate species, *S*. *bongori* [2].

**Fig 1:**
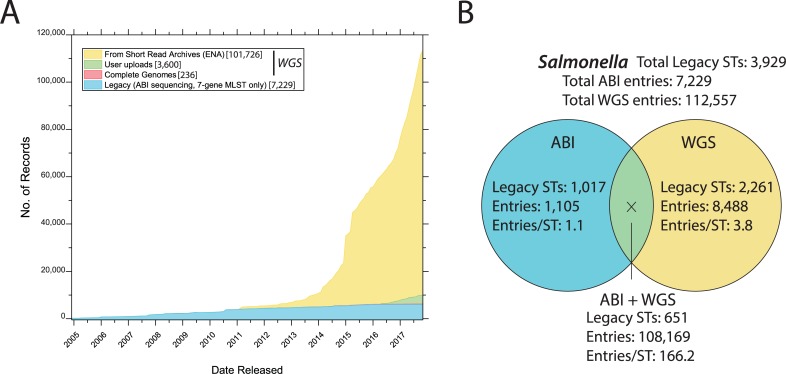
Legacy MLST STs and numbers of entries (records) in EnteroBase (http://enterobase.warwick.ac.uk). EnteroBase has performed genomic assemblies from sequence reads that were originally submitted to ENA short-read archives or directly uploaded to EnteroBase by users. EnteroBase also contains all entries with legacy MLST genotypes based on ABI sequencing that were originally submitted to the now defunct legacy MLST website. (A) Historical release dates in EnteroBase for assembled *Salmonella* genomes and strains subjected to legacy ABI-based MLST. The curves indicate an exponential increase in the numbers of publicly released short reads over time versus only a linear increase in legacy entries. Genomes with a release date after 1 November 2017 were excluded, as were 3,012 sets of short reads that showed evidence of contamination or did not pass quality control after assembly. (B) A total of 3,929 STs were defined from 120,471 strains of *Salmonella* by ABI sequencing (left), by WGS (right), or by both approaches (overlap). A full list of the strains summarised in this figure is publicly available to registered EnteroBase users in the EnteroBase workspace at https://enterobase.warwick.ac.uk/species/senterica/search_strains?query=workspace:9113. Note that the number of genomes summarised here exceeds those in part (A) because we evaluated all genomes that had been assembled by 1 November 2017, including assemblies that had not yet been released, with the exception of 795 genomic assemblies in which the alleles for all seven legacy MLST loci had not been called. ABI, Applied Biosystems; ENA, European Nucleotide Archive; WGS, whole genome sequencing. For other acronyms see Box 1.

In 2012, Achtman et al. described multilocus sequence typing (MLST) (Box 1) of *S*. *enterica* subspecies *enterica* on the basis of sequences from seven housekeeping gene fragments (legacy MLST) [3]. That publication also defined 138 genetic populations called eBurst groups (eBG1 to eBG138), which consisted of single-linkage clusters of sequence types (STs) that differed sequentially by only one of the seven sequences (single-locus variants). Most isolates within each eBG shared the same serological type, i.e., they were of the same serovar. However, many serovars were associated with multiple genetically unrelated eBGs, which indicates they are polyphyletic (derived from multiple independent ancestors). That publication [3] also posed the question, ‘Will these assignments remain stable as 10,000’s of additional isolates are investigated by MLST and genomic analyses?’ The data summarised here demonstrate that these assignments are indeed extremely stable after comparing >100,000 genomes.

**Box 1. Acronyms and explanations**

MLST (multilocus sequence typing) [4]: MLST was designed to mimic multilocus enzyme electrophoresis for its ability to reveal population structures and to reduce the impact of homologous recombination on genetic distances, which resulted because each recombination event tends to introduce multiple clustered SNPs.

SNP (single nucleotide polymorphism): a measure of genetic diversity and a common result of mutations.

ST (sequence type): a combination of numbers, each denoting a unique sequence variant at a particular gene locus.

Legacy MLST: MLST based on seven housekeeping genes. First described for *Salmonella* on the basis of serovar Typhi by Kidgell et al. in 2002 [5]. Extended to all of *Salmonella* and illustrated for *S*. *enterica* subspecies I in 2012 by Achtman et al. [3].

eBG (eBurst group): a group of related STs based on single-linkage clustering, in which the distance between nodes is only one allele. EnteroBase automatically adds new related STs to the most similar eBG unless they are one or two alleles distant from a second eBG.

rMLST: MLST based on 53 genes encoding ribosomal proteins [6]. For *Salmonella*, only 51 genes are used because 2/53 have paralogous sequences elsewhere in the genome.

rST: ST based on rMLST.

reBG: eBG in which single-linkage clustering is used to identify chains or clusters of rSTs that differ by ≤2 alleles. EnteroBase automatically adds new rSTs to the most similar reBG unless they are ≤5 alleles away from a second reBG.

cgMLST (core genome MLST): MLST based on the core genome. In the case of *Salmonella*, a soft core of 3,002 genes was selected as a subset from a whole genome MLST set.

wgMLST (whole genome MLST): MLST based on a nonredundant set of genes across a species, similar to a ‘pan-genome’.

Due to the polyphyletic nature of serovars and an error rate of serotyping of at least several percent, Achtman et al. also recommended ‘phasing out the routine use of serovars, and replacing it with a classification that is based on population genetic groupings such as eBGs and STs’. This recommendation has largely been ignored. Instead, reference laboratories continue to use serovar designations, although several now simply predict the most likely serovar from a genomic sequence on the basis of MLST [7]. Alternatively, serovars are predicted from genomes with the help of software such as Sistr [8], which uses MLST-based predictions as a tiebreaker when genomic sequences are not sufficiently discriminatory.

Finally, Achtman et al. stated, ‘the evaluation of SNPs and genomic sequences from WGS takes much more time than the evaluation of paired traces from seven gene fragments… The *S*. *enterica* MLST database will probably contain data for >10,000 isolates in the near future, as do three other MLST databases today, whereas it would currently be difficult to extract information with comparable certainty from that many genomes’. These statements were correct at the time, albeit wordy. However, those interpretations and predictions were nevertheless pessimistic, backward-looking, and wrong. In particular, the number of *Salmonella* genomic sequences has been increasing exponentially since 2012 (Fig 1A), due in large part to the adoption of high throughput genomic sequencing of *Salmonella* and other pathogens by the Wellcome Trust Sanger Centre [9–15], Public Health England [7,16], the FDA [17], and PulseNet International [18]. Academics are also beginning to sequence larger numbers of bacterial genomes. However, most genomic sequences have been deposited as sets of short reads, and >100,000 short-read sets from *Salmonella* are currently available. More and more are being deposited in the public domain as genomic assemblies, but their numbers are still far fewer than the numbers in the short-read archives [19].

Talented bioinformaticians have analysed population structures or epidemiological transmission chains within a single eBG with the help of phylogenetic trees based on single nucleotide polymorphisms (SNPs) from hundreds [9,11,16,20–24] or even thousands [15] of *Salmonella* genomes. However, this approach is time consuming and resource intensive and does not scale adequately to deal with the entire diversity of *Salmonella* that is represented by >100,000 genomes. Other current approaches are also problematic. PulseNet International has recommended the use of genotypes based on whole genome MLST (wgMLST) [18], but much of the genomic diversity among closely related strains represents variability in selfish mobile genetic elements [20], which can distort phylogenetic relationships (see below). An alternative approach is to use a core genome MLST (cgMLST) scheme [25,26]. However, the strict core genome of 361 *Salmonella* genomes, in which all genes are present and intact in all genomes, consisted of only 360 genes [8].

We have pursued still another alternative, a soft core genome: in *Salmonella*, this consists of 3,002 genes (Table 1) that were found to be present in ≥98%, intact in ≥94%, and of unexceptional diversity in 3,144 representative *Salmonella* genomes [22]. Publicly accessible websites based on the software framework BigsDB [27] present cgMLST schemes and their alleles for a variety of bacterial species [26,28], but not *Salmonella*, *Escherichia*, or *Yersinia*. We therefore developed a website, EnteroBase, which scours all short-read archives for sets of Illumina short reads from *Salmonella*, *Escherichia*, *Yersinia*, *Moraxella*, and *Clostridiodes* and supports uploading short reads by registered users. It assembles and polishes genomic contigs from the short reads within 2 hours and calculates MLST assignments from those genomes at the levels of legacy MLST, ribosomal gene MLST (rMLST) [6], soft cgMLST, and wgMLST (Table 1; Box 1) [22]. EnteroBase also supports calling SNPs from up to 1,000 genomes against a reference genome as well as the graphic evaluation of genetic relationships between entries. Here, we re-examine the population structure of *Salmonella* based on the genomic contents of more than 110,000 *Salmonella* genomes in EnteroBase (Fig 1B).

**Table 1:** *Salmonella* MLST genotyping schemes offered by EnteroBase (1 November, 2017).

Legacy MLST	rMLST	cgMLST	wgMLST
7 Loci	51 Loci	3,002 Loci	21,065 Loci
Conserved housekeeping genes	Ribosomal genes	A soft subset of core genes from wgMLST	All pan-genomic coding sequences from 537 completed/representative genomes
Conserved low resolution	Highly conserved medium resolution	Variable high resolution	Ultravariable extreme resolution
Different scheme for each species	Single scheme across tree of life	Different scheme for each genus	Different scheme for each genus
STs: 3,929 eBGs: 360	rSTs: 5,454 reBGs: 337	cgSTs: 96,108	wgSTs: 112,409

Note: The composition of 334 reBGs is consistent with the composition of eBGs. Three reBGs exist that do not correspond to one or more eBGs.

## Main text

### Legacy MLST

We have recently terminated our MLST website, which supported legacy MLST based on Applied Biosystems (ABI) sequencing. This decision was triggered because rMLST and cgMLST from genomic assemblies were much more informative than legacy MLST, and only a few new entries based on ABI sequencing were being submitted in recent years (Fig 1A). All of the historical MLST data are being maintained within EnteroBase in order to allow comparisons between data based on ABI sequences and data based on genomic assemblies.

As of 1 November 2017, 651 common *Salmonella* legacy STs had been identified by both genomic and ABI sequencing (Fig 1B). A further 2,261 less common STs were only identified by genomic sequences, and 1,017 STs are currently only defined by ABI sequences (Fig 1B). The lack of genomic support for the 1,017 STs partially reflects a strong selection bias on the strains submitted to genomic sequencing, due to a focus on human disease in Europe or North America or driven by concerns about food safety in those areas. The STs defined by ABI sequences were less biased because they represented a global effort by academics plus reference laboratories and originated from a more diverse set of sources and environments. Indeed, over the last two years genomic sequencing has identified several hundred STs that were previously only known from ABI sequences, and we anticipate that over the next few years, additional historical STs will be confirmed by genomic data. However, other STs may never be confirmed because some ABI sequence-based STs represent ABI sequencing mistakes rather than rare STs. In recent years, the MLST website curators were rejecting about 50% of supposed new alleles and new STs based on ABI sequencing that were submitted by users. Similarly, we have identified multiple false allele calls produced by our own robotic and bioinformatic pipelines [29]. Due to this experience, EnteroBase only accepts draft genomes that were generated by its own pipeline and that meet minimal criteria for contig size (N50 ≥ 20 KB), genomic size (≥4 MB), and unambiguous nucleotide calls (≥97%). Of such draft genomes, 99.3% yield sequences for all seven genes in legacy MLST. We therefore distrust the validity of the 1,017 legacy STs that are currently based exclusively on ABI sequencing (Fig 1B) until they are independently confirmed by genomic sequences.

### Association between eBG and serovar redux

eBGs were defined by Achtman et al. [3] as two or more legacy STs in a linear chain or cluster of single-locus variants, or at least 10 isolates within a singleton ST without close relatives. EnteroBase automatically adds new STs to existing eBGs when they match these criteria, unless they would result in the merging of eBGs. Additional eBGs are added at sporadic intervals with the aid of semiautomated curation, for a current total of 360 legacy eBGs. Almost all eBGs are relatively uniform for serovar. However, several percent of the publicly available metadata from the short-read archives include serovar assignments that are implausible according to their eBG assignments and in some cases even differ from published data. EnteroBase therefore provides independent serovar predictions based on the predominant serovar within each eBG and also provides the serovar predicted by Sistr [8]. The two predictions are largely congruent. However, this approach enshrines the current composition of eBGs and ignores the existence of rare exceptions. We therefore tested the stability of eBG composition by comparing them with genetic clusters defined by a totally independent set of genes, namely rMLST [6,26], based on 51 genes that encode ribosomal proteins (Table 1).

**Fig 2:**
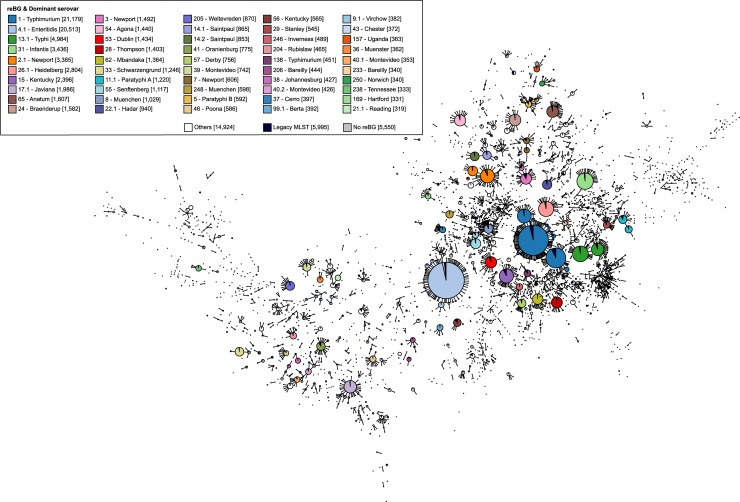
Correspondence between eBGs from legacy MLST and reBGs from rMLST. The figure shows a GrapeTree clustering (MSTreeV2) [31] of 3,929 MLST STs from 118,391 *Salmonella* strains in EnteroBase. Each node corresponds to a single legacy ST, with diameter scaled to the number of strains. Strains whose ST assignments were based on legacy ABI sequencing are coloured black and strains whose ST assignments were based on genomic assemblies are indicated by unique colours for the 50 most prevalent reBGs. The colours, reBG designations, dominant serovar, and numbers of genomic assemblies are indicated in the key (top left). Lines connect nodes that are single-locus variants. The GrapeTree shows that most reBGs correspond to a single eBG cluster of single-locus variants of legacy STs (e.g., eBG1 corresponds to reBG1), but others correspond to subclusters (e.g., eBG14 includes reBG14.1, reBG14.2, and reBG14.3). This correspondence between eBG and reBG assignments was 1:1 for 243 eBGs/reBGs. In other consistent cases, 24 eBGs split into multiple related reBGs and 13 reBGs each encompass multiple related eBGs. An interactive version of the figure is available to registered EnteroBase users at http://enterobase.warwick.ac.uk/ms_tree?tree_id=9123. A full list of strains included is available on EnteroBase (https://enterobase.warwick.ac.uk/species/senterica/search_strains?query=workspace:9113).

It might have been anticipated that 51 genes would have resulted in much higher resolution than seven genes, but the ribosomal genes are more conserved than housekeeping genes. As a result, legacy MLST of the genomes in EnteroBase defined 2,912 STs (Fig 1) and rMLST defined less than twice as many, namely 5,454 rSTs (Table 1). EnteroBase also defines reBGs as clusters of rSTs that differ in stepwise fashion by either one or two alleles. Almost all of the reBG assignments were consistent between legacy MLST and rMLST (Fig 2), with an Adjusted Rand similarity index [30] of 0.992 for the eBG and reBG assignments of STs and rSTs among 101,254 genomes (Table 2). In general, reBGs were also consistent with serovar assignments. For example, most eBG14 isolates are serovar Saintpaul, but several are serovar Haifa, which differs at the H1 flagellar antigen. eBG14 splits into reBG14.1 and reBG14.2, both of which are pure Saintpaul, as well as reBG14.3, which contains all the Haifa variants plus some Saintpaul strains. This consistent assignment to eBGs and reBGs by independent genes provide very strong evidence that almost all eBGs/reBGs represent natural genetic populations within all of *Salmonella* and can be used to predict serovar with fewer mistakes than is currently possible with serotyping.

**Table 2:** Concordance between eBG and reBG assignments among 101,254 ST and rST assignments from genomic sequences.

	reBG		
eBG	Consistent	Inconsistent	Wallace Coefficient
Consistent	405,908,145	3,852,721	0.99
Inconsistent	2,538,544	4,713,836,221	
Wallace Coefficient	0.993		

Wallace Coefficient: Adjusted Wallace Coefficient [32]. Adjusted Rand Index [30] for all data: 0.992.

We note that five exceptional sets of comparisons between eBG and reBG composition yielded conflicting clustering (Table 3). A closer examination of these five sets of conflicts with cgMLST identified complicated clustering patterns that are suggestive of the partial disruption of individual genetic populations by homologous recombination.

**Table 3:** Conflicts between eBG and reBG assignments.

Conflict	eBG	reBG (Number of Entries)	Serovar(s)
1 (both wrong)	2	2.1 (3,237), 2.2 (10)	Newport
	3	2.1 (65), 3 (1,422)	Newport
2 (both wrong)	4	4.1 (19,605), 4.2 (16), 4.3 (39), 4.4 (13), 4.5 (20)	Enteritidis/Gallinarum/Pullorum
	93	4.1 (101)	Enteritidis/Hato
3 (both wrong)	10	10.1 (67), 10.2 (15)	IIIb 61:l,v:1,5,7:[z57]
	126	10.2 (6)	IIIb 47:k:z35
	224	10.2 (28)	IIIb 38:(k):z35:[z56]
	253	10.2 (2), 253 (82)	IIIb 16:z10:e,n,[x],z15
4 (both wrong)	12	12 (266), 21.2 (315)	Brandenburg, Sandiego^1^
	21	21.1 (315), 21.2 (209)	Reading, Sandiego^1^
5 (undecided)	384.1	384 (5)	Grumpensis
	384.2	384 (8), 384.2 (9)	Telelkebir

^1^reBG12 were Brandenburg (4,[5],12:l,v:e,n,z15), reBG21.1 were Reading (1,4,[5],12:e,h:1,2), and reBG21.2 were Sandiego (1,4,[5],12:e,h:e,n,z15).

### Higher order clusters

**Fig 3:**
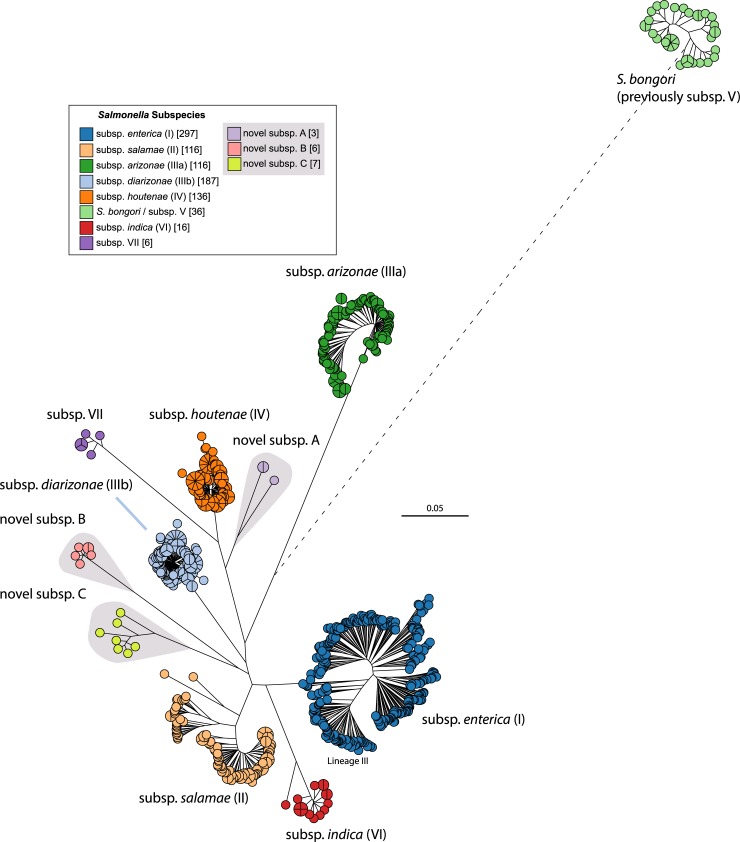
Grapetree [31] representation of a maximum-likelihood phylogeny of core SNPs from 926 representative genomes of *S*. *enterica* plus *S*. *bongori*. The dataset includes one representative genome from each reBG in *S*. *enterica* subspecies *enterica* and one representative genome from each rST in the other subspecies and *S*. *bongori*. Branches less than 0.001 substitutions per site were collapsed for clarity, whereas the branch to *S*. *bongori* (dotted line) was arbitrarily shortened to 0.4. Nodes at the tips of branches were coloured by subspecies/species, as indicated in the key. The tree indicates the likely existence of at least three genetically distinct, novel subspecies, labelled novel subsp. A through novel subsp. C. Scale bar at 02:00 in substitutions per site. A full list of strains containing the inferred species and subspecies according to this figure is available at https://enterobase.warwick.ac.uk/species/senterica/search_strains?query=workspace:9641.

Phylogenetic analyses of >100,000 genomes are currently very difficult, and it would be simpler to perform phylogenetic analyses on a representative subset that is more tractable, for example, representatives of all the diversity revealed by rMLST. Fig 3 shows a maximum-likelihood tree of core SNPs from one representative genome from each of the 297 reBGs in subspecies *enterica* plus one representative from each of 593 other rSTs in *S*. *enterica* and 36 in *S*. *bongori*. The results clearly delineate *S*. *bongori* as well as all seven known subspecies within *S*. *enterica*. They also indicate that at least three more undefined subspecies exist within *Salmonella*, which we have temporarily named novel subspecies A, B, and C, pending confirmation by standard taxonomical techniques.

Elsewhere [22], in a second analysis dedicated to subsp. *enterica* itself, we calculated a maximum-likelihood tree from 2,964 genomes, one from each of the rSTs found within the first 50,000 assembled genomes. That tree revealed the existence of 48 ‘super-lineages’ within subspecies *enterica*, a higher level of population structure than that of eBGs. One of the super-lineages, the Para C Lineage, included reBGs 6 (Choleraesuis; a swine pathogen), 20.1 (Paratyphi C; human specific), and 20.2 (Typhisuis; pigs) as well as the single isolate in rST27817 (Lomita; a very rare serovar). That analysis showed that Paratyphi C was implicated in historical invasive human disease in Europe 800 years ago [22] and subsequently in Mexico in 1545 [33], and dated the time to the most recent common ancestor of reBGs 6, 20.1, and 20.2 to about 4,000 years ago. This analysis warrants further investigations of the properties of the other super-lineages.

### Epidemiology: wgMLST versus cgMLST

The literature consists of multiple conflicting claims regarding the optimal methods for pursuing fine-scale epidemiology and transmission chains. PulseNet International now recommends wgMLST as the preferred tool for fine-scale epidemiology, replacing pulsed field gel electrophoresis (PFGE), which was previously its primary tool for this purpose [18]. EnteroBase implements a wgMLST scheme for *Salmonella* based on 21,065 orthologs from 537 complete genomes. This scheme was invaluable for defining the soft core cgMLST genome of 3,002 genes that EnteroBase uses. However, we do not recommend wgMLST for fine-scale epidemiology for several reasons. Firstly, *Salmonella* has an open pan-genome, as do many other recombining bacterial genera, and the number of orthologs in an open pan-genome grows with the number of sequenced genomes. A growing pan-genome results in a continuously growing wgMLST scheme, which in turn requires continuous calling of all genotypes as the scheme grows. Continuous reannotation of databases that already include >100,000 genomes would impose very high CPU requirements simply for maintenance. Alternatively, it might be sufficient to call genotypes on the basis of a frozen scheme, as is the case with EnteroBase. However, a frozen wgMLST scheme would lack any newly imported genes encoding antimicrobial resistance and other examples of horizontal gene transfer that were not included in the starter set of genomes from which the scheme was developed.

**Fig 4:**
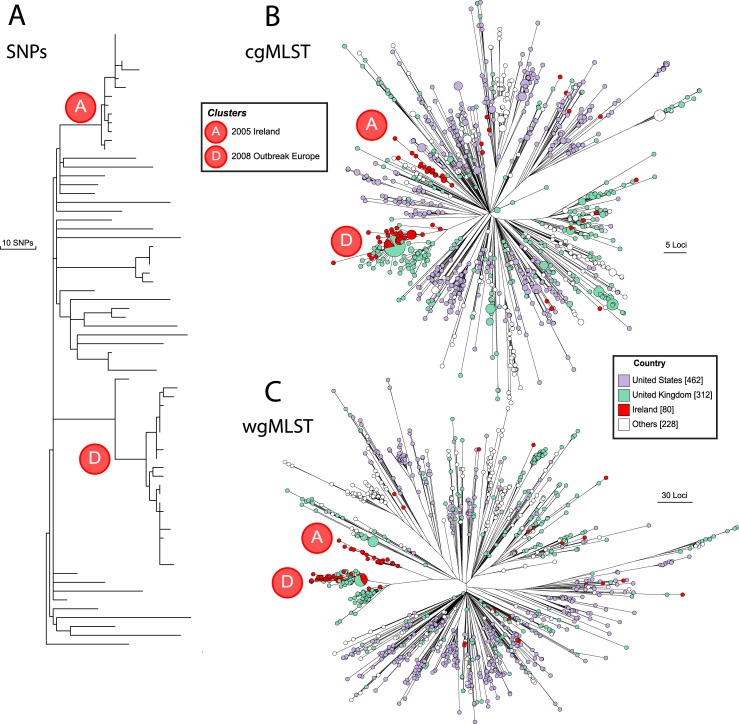
Genetic relationships according to core SNPs, cgMLST and wgMLST. (A) Maximum parsimony genealogy of 73 genomes of serovar Agona based on 846 nonhomoplastic, nonrecombinant, nonmobile, nonrepetitive core SNPs. Modified from Fig 1 of Zhou et al. [20]. Groups A and D (key) refer to the subgroups labelled A2 and A1, and D1 and D2, respectively, in that figure. An interactive version of this phylogram is available to EnteroBase users (http://enterobase.warwick.ac.uk/phylo_tree?tree_id=10290). (B) GrapeTree [31] of cgMLST (3,002 loci) from 1,082 Agona genomes in EnteroBase, consisting of all Agona genomes assembled by EnteroBase from the ENA short-read archives, including the genomes in part A plus additional genomes from the Irish 2008 outbreak. An interactive version of this tree is available at http://enterobase.warwick.ac.uk/ms_tree?tree_id=9946. The entire set of genomes and all its metadata and genotyping results are available to registered EnteroBase users at http://enterobase.warwick.ac.uk/species/senterica/search_strains?query=workspace:12810. Scale bar for 5 loci at right. (C) GrapeTree of wgMLST (21,065 loci) of the same genomes as in part B. Scale bar for 30 loci at the right. An interactive version of this tree is available at http://enterobase.warwick.ac.uk/ms_tree?tree_id=9947. Parts B and C are colour coded by country of isolation. Additional metadata such as year of isolation or source of isolation can be investigated in the interactive versions.

The accessory genome is extremely volatile due to the repeated acquisition and loss of mobile, selfish DNA within individual strains [20–22]. Thus, the primary phylogenetic signals in a wgMLST scheme are related to presence or absence of genes, and their evolutionary dynamics are currently much less well understood than are those of nucleotide diversification. To illustrate this phenomenon, we revisit serovar Agona, which caused a fast food-associated outbreak of human gastroenteritis in Ireland and the United Kingdom in 2008 [20]. The outbreak was caused by inadequately sterilised meat products due to a defective autoclave in a meat processing factory. PFGE analyses falsely indicated close relationships between the Agona from the food outbreak and other Agona from domesticated animals in Ireland in 2005. Furthermore, PFGE falsely indicated that Agona from the wastewater in the food plant were less closely related to the outbreak strain and that isolates from humans in Ireland in 2005 were only distantly related to the strains from domesticated animals. However, the PFGE patterns reflected the independent sequential acquisition by the two groups of strains of two bacterial prophages rather than changes in core restriction sites. According to core genome SNPs, the outbreak strains and the strains from the factory’s wastewater (group D) were very closely related but distant from the 2005 isolates from domesticated animals and from humans (group A) (Fig 4A). Fig 4 also shows the results of analysis of all 1,082 Agona genomes in EnteroBase by cgMLST and wgMLST. At first glance, both phylograms look similar and are dominated by the radiation of large numbers of independent branches from a common root. However, groups A and D are totally distinct according to cgMLST (Fig 4B), similar to the core SNP phylogeny (Fig 4A), whereas they represent two related subbranches according to wgMLST (Fig 4C). We therefore recommend investigating the utility of cgMLST instead of wgMLST for epidemiological purposes because it can identify very closely related *Salmonella* from a clonal outbreak as well as slightly more distantly related isolates that might represent longer term chains of transmission.

In earlier publications, SNP typing was recommended for recognising outbreaks of salmonellosis by Public Health England [16,34] and GenomeTrakr [17]. SNP typing can be extremely powerful within an eBG, but the current existence of >20,000 genomes of each of Typhimurium or Enteritidis in EnteroBase render such an approach computationally difficult. Our experience with cgMLST until now is that it approaches core SNP typing in sensitivity. Hence, we also recommend investigating the utility of cgMLST rather than SNP typing for tracking outbreaks.

We note that the graphic tool GrapeTree (Fig 2) was designed to allow the depiction of relationships based on character data such as MLST from up to 100,000 genomes [20] and visualise the cgMLST genetic distances from all of the current Enteritidis or Typhimurium isolates in EnteroBase within minutes. GrapeTree has been implemented as a visualisation tool for EnteroBase or BigsDB (personal communication, K. Jolley) and can also be used in stand-alone mode. However, although GrapeTree can also represent SNPs (Figs 3 and 4), it is too slow to visualise all core SNPs from 20,000 genomes.

Although we recommend investigating the utility of cgMLST for epidemiological purposes, we note that cgMLST is more demanding for analyses than are legacy MLST or rMLST, because cgMLST STs often contain low levels of missing data. Draft genomes based on assemblies from short reads contain multiple contigs, i.e., multiple genes are incomplete and cannot be used for calling MLST alleles. And the missing alleles can differ between independent draft genomes of the same organism. As a result, STs of natural isolates may have identical information content but differing missing data and will be assigned differing ST designations. This problem is not generally appreciated. However, EnteroBase includes approaches that minimise the missing data problem. Entries that only differ through missing data are collapsed into a single node by GrapeTree, and missing data are ignored for the calculations of genetic distances. We will soon release a form of hierarchical clustering within EnteroBase that ignores missing data and allows the recognition of natural groupings of isolates at any desired level of resolution within the cgMLST scheme, ranging from the very few loci/SNPs that distinguish isolates during a chain of transmission through to the large number that distinguish full species.

In summary, eBGs represent natural populations, and we continue to recommend that they should replace serotyping. The data also indicate that multiple additional subspecies of *Salmonella* may exist, and we recommend that epidemiologists test the abilities of cgMLST for tracing chains of disease transmission.

## Significance

Until now, it has been necessary to use a plethora of techniques to work with the genetic diversity of *Salmonella*. EnteroBase now provides a one-stop solution to analyses at all scales, ranging from the genus level (Fig 3) down to epidemiological tracing (Fig 4). EnteroBase also provides comparable opportunities for the other genera it supports, which are currently *Escherichia*, *Yersinia*, and *Clostridiodes*. Within these genera, EnteroBase provides users with the opportunity to quickly identify the genetic relatives of the bacteria whose genomes they have recently sequenced within the framework of the currently sequenced global diversity at previously unknown scales (>100,000 *Salmonella* genomes; >50,000 *Escherichia* genomes). Finally, as demonstrated here, EnteroBase supports teamwork through the facile sharing of work spaces and trees with other selected users and with the general public.

## Online resources

EnteroBase: http://enterobase.warwick.ac.uk.
